# Impact of the Adipokine Adiponectin and the Hepatokine Fetuin-A on the Development of Type 2 Diabetes: Prospective Cohort- and Cross-Sectional Phenotyping Studies

**DOI:** 10.1371/journal.pone.0092238

**Published:** 2014-03-18

**Authors:** Norbert Stefan, Qi Sun, Andreas Fritsche, Jürgen Machann, Fritz Schick, Felicia Gerst, Charlotte Jeppesen, Hans-Georg Joost, Frank B. Hu, Heiner Boeing, Susanne Ullrich, Hans-Ulrich Häring, Matthias B. Schulze

**Affiliations:** 1 Department of Internal Medicine IV, University Hospital Tübingen, Tübingen, Germany; 2 Institute of Diabetes Research and Metabolic Diseases, Member of the German Center for Diabetes Research (DZD), Tübingen, Germany; 3 Deutsches Zentrum für Diabetesforschung (DZD), Neuherberg, München, Germany; 4 Department of Nutrition, Harvard School of Public Health, Boston, Massachusetts, United States of America; 5 Channing Division of Network Medicine, Department of Medicine, Brigham and Women's Hospital and Harvard Medical School, Boston, Massachusetts, United States of America; 6 Section on Experimental Radiology, University Hospital Tübingen, Tübingen, Germany; 7 Department of Molecular Epidemiology, German Institute of Human Nutrition Potsdam-Rehbruecke, Nuthetal, Germany; 8 Department of Pharmacology, German Institute of Human Nutrition Potsdam-Rehbruecke, Nuthetal, Germany; 9 Department of Epidemiology, Harvard School of Public Health, Boston, Massachusetts, United States of America; 10 Department of Epidemiology, German Institute of Human Nutrition Potsdam-Rehbruecke, Nuthetal, Germany; University of Catanzaro Magna Graecia, Italy

## Abstract

**Background:**

Among adipokines and hepatokines, adiponectin and fetuin-A were consistently found to predict the incidence of type 2 diabetes, both by regulating insulin sensitivity.

**Objective:**

To determine to what extent circulating adiponectin and fetuin-A are independently associated with incident type 2 diabetes in humans, and the major mechanisms involved.

**Methods:**

Relationships with incident diabetes were tested in two cohort studies: within the European Prospective Investigation into Cancer and Nutrition (EPIC)-Potsdam study (628 cases) and the Nurses' Health Study (NHS; 470 cases). Relationships with body fat compartments, insulin sensitivity and insulin secretion were studied in the Tübingen Lifestyle Intervention Program (TULIP; N = 358).

**Results:**

Circulating adiponectin and fetuin-A, independently of several confounders and of each other, associated with risk of diabetes in EPIC-Potsdam (RR for 1 SD: adiponectin: 0.45 [95% CI 0.37–0.54], fetuin-A: 1.18 [1.05–1.32]) and the NHS (0.51 [0.42–0.62], 1.35 [1.16–1.58]). Obesity measures considerably attenuated the association of adiponectin, but not of fetuin-A. Subjects with low adiponectin and concomitantly high fetuin-A had the highest risk. Whereas both proteins were independently (both p<1.8×10^−7^) associated with insulin sensitivity, circulating fetuin-A (r = −0.37, p = 0.0004), but not adiponectin, associated with insulin secretion in subjects with impaired glucose tolerance.

**Conclusions:**

We provide novel information that adiponectin and fetuin-A independently of each other associate with the diabetes risk. Furthermore, we suggest that they are involved in the development of type 2 diabetes via different mechanisms, possibly by mediating effects of their source tissues, expanded adipose tissue and nonalcoholic fatty liver.

## Introduction

Among several pathways involved in the pathogenesis of the epidemically spreading disease type 2 diabetes, an altered secretory pattern of the expanded and inflamed adipose tissue is thought to be important for the regulation of insulin sensitivity and subclinical inflammation in various tissues [1]. In this respect adiponectin has gained much attention in the past years because the circulating levels of this adipokine are not only markers of type 2 diabetes risk, but because adiponectin is strongly involved in its progression [2]. In analogy to dysregulated adipose tissue [2]–[4], there is increasing evidence that nonalcoholic fatty liver disease (NAFLD), which predictive of metabolic diseases [5]–[10], is also associated with an altered secretory pattern of proteins, which can be referred to as hepatokines, and which are both markers of the disease, and are involved in its pathophysiology [11]. Among them fetuin-A gained much attention during the recent years because of its association with type 2 diabetes and cardiovascular disease risk [12]-[17]. and its important role in the pathogenesis of insulin resistance and subclinical inflammation [18]–[23].

In the present study we now asked two questions: first, to what extent are circulating levels of these proteins related to incident type 2 diabetes independently of each other? Second, because the circulating levels of these two proteins strongly reflect the dysregulation of their source tissues, adipose tissue and liver, can they be used to estimate the contribution of expanded and inflamed adipose tissue and NAFLD to the pathogenesis of insulin resistance and impaired beta cell function?

For this we investigated associations of circulating adiponectin and fetuin-A with incident type 2 diabetes by applying a head to head comparison of these proteins in two large cohort studies, the European Prospective Investigation into Cancer and Nutrition (EPIC)-Potsdam study and the Nurses' Health Study (NHS). In addition, we studied the independent relationships of the circulating levels of these proteins with precisely measured body fat mass and distribution, liver fat content, insulin sensitivity and insulin secretion in subjects of the Tübingen Lifestyle Intervention Program (TULIP).

## Subjects and Methods

### EPIC-Potsdam study

The EPIC-Potsdam Study is part of the multi-centre prospective cohort study EPIC [24]. In Potsdam, Germany, 27,548 subjects (16,644 women and 10,904 men) were recruited from the general population between 1994 and 1998. The age range was 35–65 years in women and 40–65 years in men. The baseline examination included anthropometric measurements, a personal interview including questions about prevalent diseases, and a questionnaire about socio-demographic and lifestyle characteristics [13]. Follow-up questionnaires were sent out every 2 to 3 years to identify incident cases of type 2 diabetes. All incident cases of diabetes identified were verified by treating physicians. Biochemical measurements were carried out in a case-cohort design nested within the cohort, details of which have been published previously [13]. Briefly, we randomly selected a subcohort of 2,500 individuals of whom 2,095 were non-diabetic at baseline (based on self-report and blood glucose determinations) and had anamnestic, anthropometrical and metabolic data for analysis. Of the 849 incident diabetes cases identified in the full cohort during an average follow-up of 7 years 628 remained for analyses after similar exclusions. Informed consent was obtained from all participants, and approval was given by the Ethical Committee of the State of Brandenburg, Germany.

### NHS

A total of 121,700 registered nurses living in one of 11 populous U.S. states composed the NHS when they responded to a questionnaire inquiring about their medical history and lifestyle characteristics in 1976 [15]. In 2000–2001, 18,717 NHS participants aged 53–79 years provided blood samples. Among these participants, a prospective, nested, case-control study was conducted to examine plasma biomarkers in relation to type 2 diabetes risk. After excluding women with self-reported prevalent diabetes, cardiovascular disease, or cancer at baseline, 470 cases of type 2 diabetes cases from the date of blood draw through June 2006 were prospectively identified and confirmed. Risk-set sampling was used to randomly select one control for each case from the rest of population who remained free of diabetes when the case was diagnosed; the probability of being selected as a control is proportional to the length of follow-up. Cases and controls were further matched for age at blood draw (61 year), date of blood draw (63 months), fasting status (fast for 8 h or not), and race (white or other races) [15]. The study protocol was approved by the institutional review board of the Brigham and Women's Hospital and the Human Subjects Committee Review Board of Harvard School of Public Health.

### TULIP

A total of 358 Caucasians, who participated in the Tübingen Lifestyle Intervention Program (TULIP) [24], were included in the present analyses because they fulfilled at least one of the following criteria: a family history of type 2 diabetes, a BMI>27 kg/m^2^, previous diagnosis of impaired glucose tolerance or gestational diabetes. Informed written consent from subjects participating in the Tübingen studies was obtained and the Ethical Committee of the University of Tübingen, Germany had approved the protocols.

Anthropometrics and metabolic parameters were measured as previously described [13], [25], [26]. Glucose tolerance was determined according to the 1997 World Health Organization diagnostic criteria [27]. Insulin sensitivity from the OGTT was estimated as proposed by Matsuda and DeFronzo [28]. In a subgroup (N = 244) insulin sensitivity was also measured during a euglycemic, hyperinsulinemic clamp [26]. The insulinogenic index, a precise estimate of glucose-induced insulin secretion, was assessed from the OGTT as follows: (insulin at 30 min-insulin at 0 min)/(glucose at 30 min-glucose at 0 min).

### Measurement of adiponectin and fetuin-A

In the EPIC-Potsdam study and in the TULIP study adiponectin levels were determined with enzyme-linked immunosorbent assays (ELISA, Linco Research, Inc., St Charles, MO). Fetuin-A levels were measured using an immunoturbidimetric method (BioVendor Laboratory Medicine, Modreci, Czech Republic). In the NHS, both, adiponectin and fetuin-A levels were measured by enzyme immunoassays from R&D Systems (Minneapolis, MN).

### Statistical analyses

In the EPIC Study and the NHS adiponectin and fetuin-A levels were categorized into quintiles based on subcohort or control participants. Hazard ratios as a measure of relative risk (RR) were computed using a weighted Cox proportional hazards model in EPIC-Potsdam, modified for the case-cohort design according to the Prentice method. Age was the underlying time variable in the counting processes, with entry defined as the subjects' age at the time of recruitment and exit defined as age at the diagnosis of diabetes, or censoring. In NHS, odds ratios were calculated using unconditional logistic regression to evaluate strength of associations [15].

We computed RRs/ORs for each quintile of adiponectin and fetuin-A compared with the lowest quintile. The significance of linear trends across quintiles of adiponectin and fetuin-A was tested by assigning each participant the median value for the quintile and modeling this value as a continuous variable. Because this analysis indicated no department from linearity, we also considered adiponectin and fetuin-A as continuous variable estimating the RR/OR associated with an increment of 1 SD. We used information on covariates obtained from the baseline examination in multivariate analyses, namely sex, education, physical activity, smoking, and alcohol intake. Analyses in NHS were further adjusted for other matching factors beyond age (race, fasting status, time of blood drawing), as well as for body mass index (BMI) and waist circumference.

In the TULIP study, data are given as means±SE. Data that were not normally distributed (e.g. liver fat, insulin sensitivity, body fat distribution; Shapiro-Wilk W test) were logarithmically transformed. A p-value≤0.05 was considered statistically significant.

## Results

### Association of circulating adiponectin and fetuin-A with diabetes incidence in the EPIC-Potsdam Study and the NHS

The adjusted RRs/ORs of type 2 diabetes for quintiles of fetuin-A and adiponectin in the EPIC-Potsdam study and the NHS are shown in table 1. Higher levels of fetuin-A were associated with an increased risk of diabetes, independent of adiponectin (RR/OR comparing extreme quintiles: EPIC-Potsdam: 1.23 [95% CI: 0.88–1.72], p for trend <0.01; NHS: 2.05 [1.24–3.37]). Contrary, higher adiponectin levels were associated with lower risk of diabetes in both cohorts (RR/OR comparing extreme quintiles: EPIC-Potsdam: 0.20 [0.13–0.30]; NHS: 0.18 [0.10–0.32]). Considering fetuin-A and adiponectin as continuous variables (per 1 SD increment), revealed a similar picture with consistent inverse associations for adiponectin and positive associations for fetuin-A (Table 2). Adjustment for BMI and waist circumference attenuated the associations of adiponectin (by 29% in EPIC-Potsdam and 28% in NHS). However, the association of fetuin-A with diabetes risk was largely unaffected by adjustment for BMI and waist circumference (Table 2).

**Table 1:** Risk of type 2 diabetes by quintiles of plasma adiponectin and fetuin-A.

	Quintiles of biomarker					P Trend
	1	2	3	4	5	
**EPIC-Potsdam** *						
*Adiponectin*						
Median (μg/ml)	3.83	6.23	8.13	9.96	13.4	
Cases	253	148	106	77	44	
Relative Risk (95% CI)	1.00	0.49	0.37	0.34	0.20	<0.0001
		(0.36, 0.66)	(0.27, 0.51)	(0.24, 0.48)	(0.13, 0.30)	
						
*Fetuin-A*						
Median (μg/ml)	178	221	249	280	323	
Cases	102	100	124	137	165	
Relative Risk (95% CI)	1.00	0.68	0.96	1.21	1.23	0.0085
		(0.47, 0.99)	(0.68, 1.37)	(0.86, 1.70)	(0.88, 1.72)	
						
**Nurses' Health Study** #						
*Adiponectin*						
Median (μg/ml)	4.59	6.53	8.62	11.2	16.0	
Case/control	245/94	83/94	78/94	39/94	25/94	
Odds Ratio (95% CI)	1.00	0.34	0.40	0.23	0.18	
		(0.23, 0.52)	(0.26, 0.61)	(0.14, 0.38)	(0.10, 0.32)	<0.0001
						
*Fetuin-A*						
Median (μg/ml)	347	421	474	535	626	
Case/control	59/94	79/94	80/94	124/94	128/94	
Odds Ratio (95% CI)	1.00	1.16	1.49	2.06	2.05	0.0008
		(0.69, 1.93)	(0.89, 2.49)	(1.26, 3.36)	(1.24, 3.37)	

*Adiponectin and fetuin-A included simultaneously in the model; Adjusted for age, sex, BMI, waist circumference, education (in or no training, vocational training, technical school, or technical college or university degree), occupational activity (light, moderate, heavy), sport activity (0, 0.1–4.0, >4.0 h/week), cycling (0, 0.1–2.4, 2.5–4.9, ≥5.0 h/week), smoking (never, past, current <20 cigarettes/d, current > = 20 cigarettes/d), and alcohol intake (0, 0.1–5.0, 5.1–10.0, 10.1–20.0, 20.1–40.0, >40.0 g/d).

# Adiponectin and fetuin-A included simultaneously in the model. In addition, multivariate model was adjusted for matching factors, including age at blood draw (yrs), race (white or not), fasting status (yes, no), and time of blood drawing, as well as body mass index (kg/m2), waist circumference (cm), smoking status (current smoker, past smoker, non-smoker), physical activity (in tertiles), alcohol use (abstainer, <5.0 g/day, 5.0–14.9 g/day, ≥15.0 g/day), and education (registered nurse, bachelor, master and higher).

**Table 2:** Relative Risk of type 2 diabetes for plasma adiponectin and fetuin-A and attenuation of the risk by BMI and waist circumference.

	Adiponectin		Fetuin-A	
	RR/OR 1 SD	% change	RR/OR 1 SD	% change
				
**EPIC-Potsdam**				
Model 1 *	0.35	Ref	1.21	Ref
	(0.29, 0.41)		(1.09, 1.34)	
Model 1 + BMI	0.43	+22.9	1.19	−1.7
	(0.36, 0.52)		(1.06, 1.33)	
Model 1 + BMI + waist circumference	0.45	+28.6	1.18	−2.5
	(0.37, 0.54)		(1.05, 1.32)	
				
**Nurses' Health Study**				
Model 1 ^#^	0.40	Ref	1.32	Ref
	(0.33–0.48)		(1.14–1.53)	
Model 1 + BMI	0.47	+17.5	1.38	+4.5
	(0.39–0.57)		(1.18–1.61)	
Model 1 + BMI + waist circumference	0.51	+27.5	1.35	+2.3
	(0.42–0.62)		(1.16–1.58)	

*Relative Risk adjusted for age, sex, education (in or no training, vocational training, technical school, or technical college or university degree), occupational activity (light, moderate, heavy), sport activity (0, 0.1–4.0, >4.0 h/week), cycling (0, 0.1–2.4, 2.5–4.9, ≥5.0 h/week), smoking (never, past, current <20 cigarettes/d, current ≥20 cigarettes/d), and alcohol intake (0, 0.1–5.0, 5.1–10.0, 10.1–20.0, 20.1–40.0, >40.0 g/d).

# Odds Ratio adjusted for matching factors, including age at blood draw (yrs), race (white or not), fasting status (yes, no), and time of blood drawing, as well as smoking status (current smoker, past smoker, non-smoker), physical activity (in tertiles), alcohol use (abstainer, <5.0 g/day, 5.0–14.9 g/day, ≥15.0 g/day), and education (registered nurse, bachelor, master and higher).

**Figure 1:**
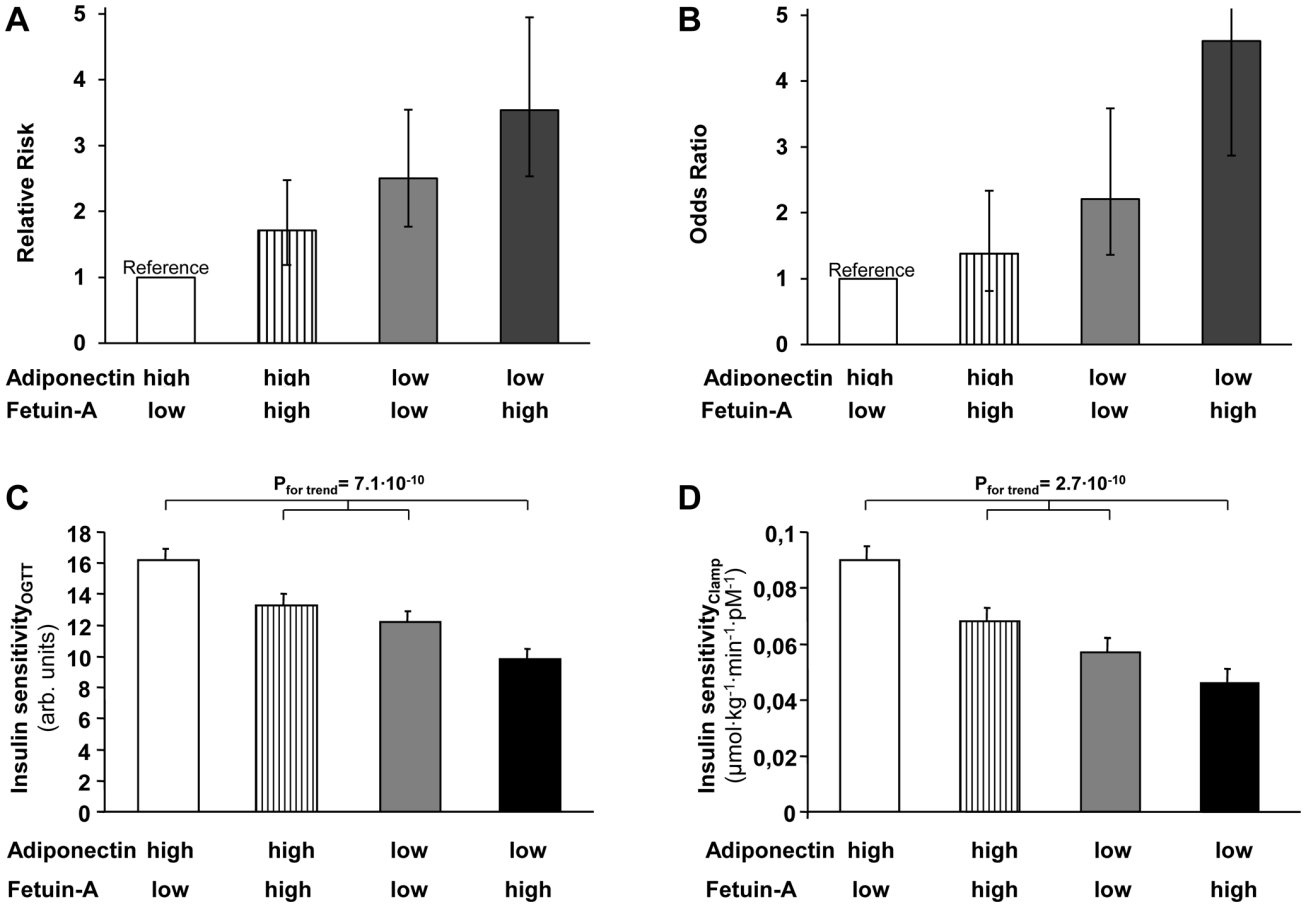
Relative risk (in the European Prospective Investigation into Cancer and Nutrition (EPIC)-Potsdam Study; Panel A) and Odds Ratio (in the Nurses' Health Study; Panel B) of type 2 diabetes for joint classifications of plasma adiponectin and fetuin-A. Groups with high/low adiponectin and fetuin-A levels were defined based on sex-specific medians. RRs were adjusted for age, sex, education, occupational activity, sport activity, cycling, smoking, alcohol intake, BMI, and waist circumference in EPIC-Potsdam and ORs for matching factors, including age at blood draw, race, fasting status, and time of blood drawing, as well as smoking status, physical activity, alcohol use, and education in the Nurses' Health Study. Relationship of circulating adiponetin and fetuin-A with insulin sensitivity estimated from the oral glucose tolerance test (OGTT; N = 358, C) and measured during the euglycemic, hyperinsulinemic clamp (N = 244, D). Subjects were divided by the medians of circulating adiponectin and fetuin-A in groups with high and low levels. P for trend after adjustment for age and sex.

We then examined the joint effect of circulating adiponectin and fetuin-A by cross classifying participants by both variables using sex-specific medians as cut-offs. The RR/OR for the combination of a high fetuin-A- and a low adiponectin level compared with the opposite extreme was 3.54 (95% CI: 2.54–4.95) in EPIC-Potsdam and 4.61 (2.87–7.38) in NHS (Figure 1, A/B). Tests for interaction were non-significant in both cohorts.

### Cross-sectional relationships in the TULIP

The 358 TULIP subjects had a mean age of 46 years and a mean BMI of 30 kg•m^−2^. To investigate relationships of circulating adiponectin and fetuin-A levels with metabolic traits we performed analyses in the total population and in subjects with normal glucose tolerance (NGT) and impaired glucose tolerance (IGT) separately (Table 1 in File S1).

#### Relationships of circulating adiponectin and fetuin-A with body fat content and distribution and with insulin sensitivity

In the total population circulating adiponectin, adjusted for age and gender, correlated negatively with BMI (r = −0.19, p = 0.0004) and with waist circumference (r = −0.26, p<0.0001). Negative correlations were also found with total body fat mass (r = −0.15, p = 0.008) and, more strongly, with visceral fat mass (r = −0.40, p<0.0001) and with liver fat content (r = −0.28, p<0.0001). Circulating fetuin-A correlated only very weakly with BMI (r = 0.11, p = 0.04) and not statistically significant with waist circumference (r = 0.10, p = 0.06), total body fat mass- (r = 0.07, p = 0.25), and visceral fat (r = 0.07, p = 0.20) mass. However, a positive correlation with liver fat content was found (r = 0.12, p = 0.04). In multivariate models including age and sex, both circulating adiponectin and fetuin-A were strongly and independently associated with insulin sensitivity estimated from the OGTT and measured during the clamp (Table 3, models 1). Inclusion of BMI and waist circumference in the models attenuated the associations of circulating adiponectin (change of the β; OGTT: −37%, clamp: −31%) on insulin sensitivity, but less so of circulating fetuin-A (OGTT: −17%, clamp: −18%) (Table 3, models 2). Similar relationships were found when these analyses were performed in subjects with and without NAFLD (OGTT: total N = 291, NAFLD = 93; clamp: total N = 203, NAFLD = 63). Here inclusion of BMI and waist circumference in the models attenuated the associations of circulating adiponectin with insulin sensitivity, particularly in subjects without NAFLD (change of the β; OGTT: −32%, clamp: −34%), while this association was only slightly attenuated or even became stronger in the smaller group of subjects with NAFLD (OGTT: −7%, clamp: +18%). The respective relationships of fetuin-A with insulin sensitivity were less strongly affected, both, in subjects without (OGTT: −12%, clamp: −13%) and with (OGTT: −6%, clamp: −4%) NAFLD. After additional inclusion of liver fat content in the models 2, the associations of adiponectin and fetuin-A with insulin sensitivity were further attenuated and adiponectin, fetuin-A and liver fat content independently determined insulin sensitivity (Table 3, models 3). When we then divided non-obese subjects (N = 207) by the median insulin sensitivity estimated by the OGTT (13.78 arb.u.) we found circulating fetuin-A (OR for 1 SD: 1.42 [95% CI 1.09–1.85]) but not circulating adiponectin (0.88 [0.77–1.01]) or hs-CRP levels (1.04 [0.98–1.11]) to predict the insulin resistant state, independently of age, sex, BMI and waist circumference. Similar relationships were found when NGT and IGT were analyzed separately (data not shown).

**Table 3:** Determinants of insulin sensitivity in multivariate regression models in TULIP.

	Insulin sensitivity_OGTT_			Insulin sensitivity_Clamp_		
Covariates	Estimate±SE	F-value	p	Estimate±SE	F-value	p
**Models 1**						
Female sex	0.04±0.03	1.3	0.25	0.003±0.04	0.0	0.93
Age	−0.29±0.11	6.8	0.01	−0.31±0.13	6.0	0.015
Adiponectin levels	0.38±0.07	30.4	<0.0001	0.52±0.08	43.5	<0.0001
Fetuin-A levels	−0.85±0.16	28.4	<0.0001	−0.72±0.18	16.1	<0.0001
						
**Models 2**						
Female sex	−0.01±0.04	0.24	0.63	−0.03±0.04	0.5	0.48
Age	−0.17±0.10	2.6	0.11	−0.17±0.11	2.3	0.13
Adiponectin levels	0.24±0.06	14.2	0.0002	0.36±0.07	25.3	<0.0001
Fetuin-A levels	−0.70±0.14	23.8	<0.0001	−0.59±0.16	14.4	0.0002
BMI	−−0.54±0.31	3.0	0.08	−1.05±0.33	10.1	0.002
Waist circumference	−1.16±0.43	7.4	0.007	−0.68±0.45	2.3	0.13
						
**Models 3** *						
Female sex	−0.004±0.04	0.01	0.91	−0.03±0.04	0.6	0.44
Age	−0.09±0.10	0.7	0.39	−0.007±0.11	0.004	0.95
Adiponectin levels	0.17±0.07	6.5	0.01	0.27±0.07	14.2	0.0002
Fetuin-A levels	−0.69±0.15	22.9	<0.0001	−0.48±0.16	9.2	0.003
BMI	−0.91±0.34	7.2	0.008	−0.91±0.36	6.4	0.01
Waist circumference	0.13±0.43	0.07	0.79	−0.06±0.50	0.02	0.90
Liver fat content	−0.20±0.03	52.0	<0.0001	−0.20±0.03	48.2	<0.0001

*N = 291 for insulin sensitivity_OGTT_ and N = 203 for insulin sensitivity_Clamp_.

When we divided participants by the medians of circulating adiponectin and fetuin-A levels, individuals with low adiponectin and high fetuin-A levels had the lowest insulin sensitivity compared to the other groups (Figure 1, C/D).

#### Relationships of circulating adiponectin and fetuin-A with insulin secretion

**Figure 2:**
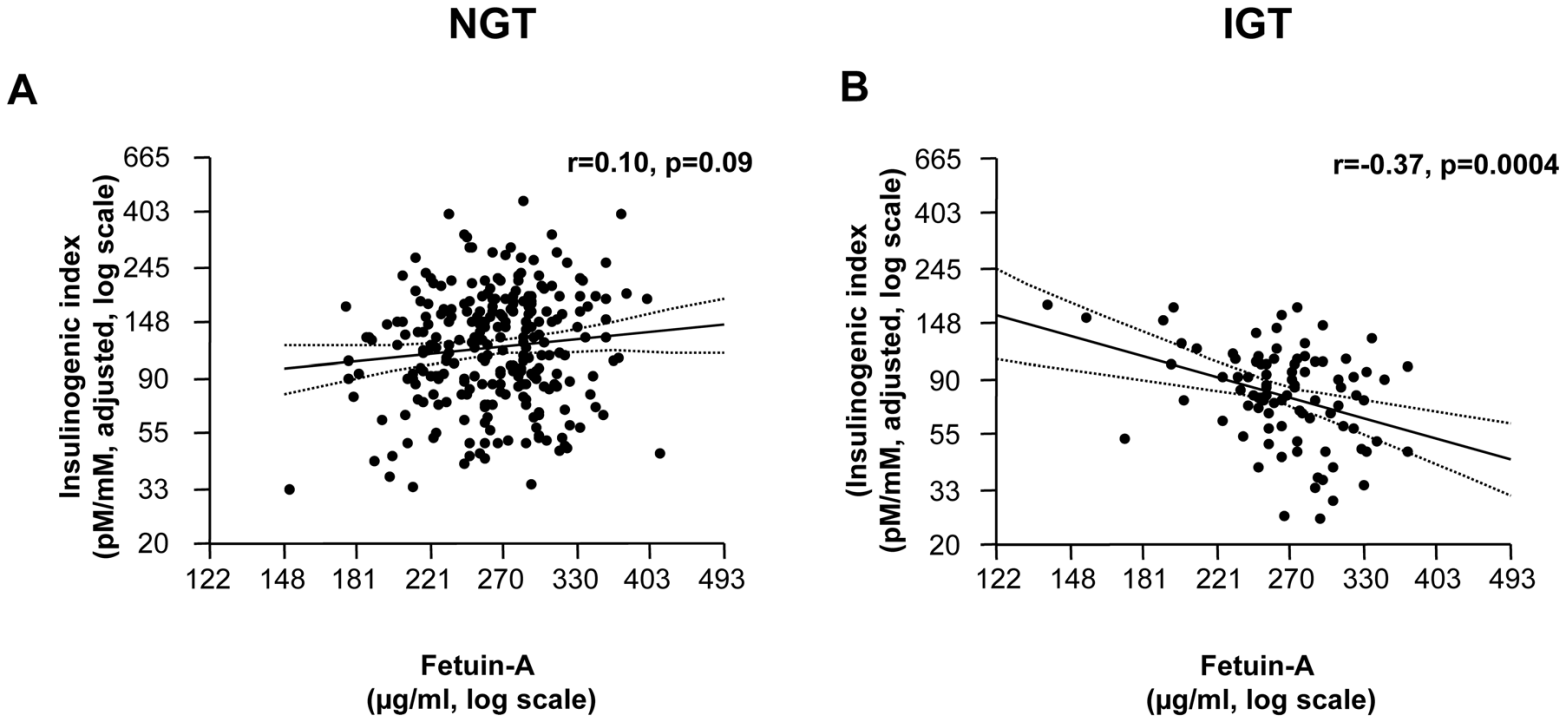
Relationship of circulating fetuin-A with the insulinogenic index, adjusted for age, sex and insulin sensitivity estimated from the oral glucose tolerance test, in subjects with normal glucose tolerance (N = 267; A) and impaired glucose tolerance (N = 91, B).

We next investigated whether circulating fetuin-A may be associated with glucose-induced insulin secretion in humans. In the total population circulating fetuin-A did not correlate with the insulinogenic index (r<0.01 p = 0.99) when adjusted for age, gender and insulin sensitivity measured during the OGTT. However, this association depended on glucose tolerance status: while fetuin-A did not correlate with the insulinogenic index in subjects with NGT, a strong negative correlation between fetuin-A levels and the insulinogenic index was found in subjects with IGT (r = −0.37, p = 0.0004) (p for interaction = 0.024) (Figure 2, A/B). No significant relationships were found for circulating adiponectin with the adjusted insulinogenic index (all p>0.055).

## Discussion

During the last decade much effort has been made to identify important pathways involved in the natural history of type 2 diabetes. Thereby, several candidates were described, predominantly based on animal and on in-vitro studies [29]–[31]. However, often it was not possible to prove these pathways to be of high relevance for human metabolism. In human studies, on the other hand, several blood, genetic or phenotypic markers were found to predict incident type 2 diabetes [1]–[4]. Nevertheless, no precise mechanisms of action for several of these parameters are known and/or their predictive effect on the development of type 2 diabetes was either small or absent, which so far limits their potential in the prevention and the treatment of the disease.

Because these limitations largely do not apply to the adipokine adiponectin and the hepatokine fetuin-A, we here investigated to what extent circulating adiponectin and fetuin-A determine incident type 2 diabetes, independently of each other. Towards this aim, we first chose an epidemiological approach and investigated the associations of circulating adiponectin and fetuin-A with incident type 2 diabetes by applying a head to head comparison of these proteins in two large cohort studies, the EPIC-Potsdam study and the NHS. In both studies we found that circulating adiponectin and fetuin-A were associated with risk of incident diabetes, independently of several confounders, and of each other. The consistency of the association suggests that it might be generalizable to healthy populations, at least to those with Caucasian origin. Because the strength of association of adiponectinemia, but not of circulating fetuin-A, was considerably attenuated after accounting for estimates of overall and visceral obesity, our data support that the adiponectin levels confer at least in part the effect of obesity on the type 2 diabetes risk. In contrast, the association of fetuin-A with diabetes risk does not appear to considerably depend on body fatness. Rather the available information from our and other studies suggest that the increase in circulating fetuin-A and the resulting decrease in insulin sensitivity may be a result of inflamed NAFLD [11], [32]–[35].

We then focused on the relationship of both circulating proteins with anthropometrics and metabolic traits in precisely phenotype subjects of TULIP. We confirmed the strong correlations of adiponecinemia with measures of body fat mass and distribution in these subjects as well as the absence of such relationships for fetuin-A levels. Based on the known properties of adiponectin and fetuin-A to regulate insulin sensitivity, we confirmed that the circulating levels of these proteins were independently of each other associated with insulin sensitivity, estimated from the OGTT or measured by a euglycemic, hyperinsulinemic clamp. In agreement with the findings from the EPIC-Potsdam study and the NHS, the relationship of circulating adiponectin, but not of fetuin-A, was considerably attenuated after accounting for measurements of body fat content and distribution. Consequently we asked the question whether circulating fetuin-A may be a better predictor of insulin sensitivity than circulating adiponectin in subjects who are non-obese and could confirm this hypothesis in our study.

Having found strong independent associations of circulating adiponectin and fetuin-A, the two proteins that regulate insulin sensitivity, on the diabetes risk, we then asked whether they may differentially impact on insulin secretion, and thereby have distinct effects in the pathogenesis of type 2 diabetes. For adiponectin we have previously shown that this protein does not influence glucose-induced insulin secretion in humans [36]. In the present study we could show that fetuin-A levels are not associated with insulin secretion in our subjects. Based on the knowledge that subjects with IGT have an impaired beta cell function [37], [38], we then tested the hypothesis that fetuin-A is particularly relevant specifically in this population that is at very high risk for the disease. Indeed, when we separated the individuals in those with NGT and IGT, a strong negative relationship of fetuin-A with insulin secretion was found in subjects with IGT.

What is the relevance of our data for clinicians and researchers? Because the relative risk of incident diabetes was much higher for the combination of a high fetuin-A- and a low adiponectin level, than for the single circulating level of each protein, it may be important for clinicians to measure both proteins when it comes to the prediction of the risk of future type 2 diabetes. Whether fetuin-A and adiponectin improve prediction of diabetes risk beyond waist circumference and other classical risk factors remains, however, uncertain. Furthermore, fetuin-A may become an important determinant of insulin resistant states, particularly in non-obese subjects where adiponectin and hs-CRP levels lost their strong predictive power in our study.

For researchers we provide novel information that adiponectin and fetuin-A are independently involved in the pathogenesis of type 2 diabetes. Both proteins impact on the development of the disease predominantly be the regulation of subclinical inflammation. Furthermore, we have support for the hypothesis that they mediate the effects of their source tissues, expanded adipose tissue and inflamed nonalcoholic fatty liver on glucose metabolism and cardiovascular disease. In addition, we provide explorative information about a putatively newly identified cross-talk of the liver with the endocrine function of the pancreas. Whether there are direct effects of fetuin-A on signalling cascades in beta cells or whether fetuin-A induces a chronic pro-inflammatory process in the human islets needs to be investigated in future studies.

Some possible limitations of our findings have to be considered. The potential of residual confounding applies to our study as it does to observational studies in general. We adjusted for a large variety of known risk factors. Although fetuin-A and adiponectin remained significantly associated with diabetes risk, we cannot rule out that other unmeasured factors or that imprecision in the measurement of covariates explain this observation. Also, we only had a single blood drawing which might have introduced random measurement errors in determining fetuin-A and adiponectin. The lack of repeated measurements may have led to an underestimation of the observed associations. In conclusion, our findings support that the adipokine adiponectin and the hepatokine fetuin-A are reliable predictors of incident type 2 diabetes and insulin resistance, and that they are strongly and independently of each other involved in their pathogenesis. Moreover, we could provide indirect support that adiponectin mediates, at least in part, the impact of dysregulated and expanded visceral fat on metabolism, whereas increased fetuin-A levels are largely a result of NAFLD. Finally, we provided novel exploratory information that fetuin-A may play a role in the pathogenesis of type 2 diabetes by affecting insulin secretion.

## Supporting Information

Table S1 **Characteristics of the TULIP study participants.**
(DOC)
