# Supplementary material for: Impact of the Adipokine Adiponectin and the Hepatokine Fetuin-A on the Development of Type 2 Diabetes: Prospective Cohort- and Cross-Sectional Phenotyping Studies
Source: PLoS One. 2014 Mar 18;9(3):e92238. doi: 10.1371/journal.pone.0092238 (PMC3958485; doi:10.1371/journal.pone.0092238)
Supplement: Table S1 — Characteristics of the TULIP study participants. (DOC) [file pone.0092238.s001.doc]

**Table S1** Characteristics of the TULIP study participants

|  | **All** | **NGT** | **IGT** | **p**  **(NGT vs IGT)** |
| --- | --- | --- | --- | --- |
| **Characteristics** |
| N | 358 | 267 | 91 |  |
| Gender (females / males) | 226 / 132 | 165 / 102 | 61 / 30 | 0.37 |
| Age (years) | 46 ± 1 | 45 ± 1 | 49 ± 1 | 0.003 |
| Body weight (kg) | 87.6 ± 1.0 | 87.7 ± 1.2 | 87.4 ± 1.8 | 0.97 |
| BMI (kg . m^-2^) | 30.0 ± 0.3 | 29.7 ± 0.3 | 30.9 ± 0.6 | 0.10 |
| Waist circumference (cm) | 97.2 ± 0.74 | 96.4 ± 0.9 | 99.2 ± 1.4 | 0.09 |
| Total body fat (kg)* | 26.5 ± 0.6 | 26.1 ± 0.7 | 27.7 ± 1.3 | 0.18 |
| Visceral fat (kg)* | 2.97 ± 0.11 | 2.79 ± 0.12 | 3.53 ± 0.23 | 0.003 |
| Liver fat (%)* | 5.99 ± 0.39 | 4.76 ± 0.36 | 9.55 ± 1.01 | <0.0001 |
| Fasting glucose (mM) | 5.27 ± 0.03 | 5.16± 0.03 | 5.55 ± 0.07 | <0.0001 |
| 2 h glucose (mM) | 6.92 ± 0.09 | 6.15 ± 0.06 | 9.15 ± 0.11 | <0.0001 |
| Insulin sensitivity_OGTT_ (AU) | 12.84 ± 0.38 | 14.06 ± 0.45 | 9.28 ± 0.49 | <0.0001 |
| ISI_Clamp_ (μmol·kg^-1^·min^-1^·pM^-1^)^§^ | 0.065 ± 0.002 | 0.071 ± 0.003 | 0.048 ± 0.003 | <0.0001 |
| Insulinogenic index (pM/mM)^#^ | 128.6 ± 4.4 | 140.9 ± 5.3 | 92.6 ± 6.1 | <0.0001 |
| HbA1c (%) | 5.62 ± 0.02 | 5.54 ± 0.02 | 5.82 ± 0.05 | <0.0001 |
| hs-CRP (mg/dl) | 0.24 ± 0.02 | 0.22 ± 0.02 | 0.29 ± 0.04 | 0.03 |
| Adiponectin (μg/ml) | 13.77 ± 0.35 | 14.24 ± 0.43 | 12.44 ± 0.51 | 0.08 |
| Fetuin-A (μg/ml) | 269 ± 3 | 268 ± 3 | 273 ± 6 | 0.63 |

Values represent means ± SE (standard error). ISI, insulin sensitivity index; hs-CRP, high-sensitive C-reactive protein. ^#^ available in 356 subjects, *available in 304 (liver fat in 291) subjects, ^§^available in 244 subjects
